# The Early Result of Whole Pelvic Radiotherapy and Stereotactic Body Radiotherapy Boost for High-Risk Localized Prostate Cancer

**DOI:** 10.3389/fonc.2014.00278

**Published:** 2014-10-31

**Authors:** Yu-Wei Lin, Li-Ching Lin, Kuei-Li Lin

**Affiliations:** ^1^Department of Radiation Oncology, Chi Mei Medical Center, Tainan, Taiwan; ^2^Institute of Biomedical Sciences, National Sun Yat-sen University, Kaohsiung, Taiwan; ^3^The School of Medicine, Kaohsiung Medical University, Kaohsiung, Taiwan; ^4^School of Medicine, Taipei Medical University, Taipei, Taiwan

**Keywords:** prostate cancer, high-risk prostate cancer, stereotactic body, radiotherapy, SBRT boost, whole-pelvis radiotherapy, CyberKnife, Rapidarc

## Abstract

**Purpose**: The rationale for hypofractionated radiotherapy in the treatment of prostate cancer is based on the modern understanding of radiobiology and advances in stereotactic body radiotherapy (SBRT) techniques. Whole-pelvis irradiation combined with SBRT boost for high-risk prostate cancer might escalate biologically effective dose without increasing toxicity. Here, we report our 4-year results of SBRT boost for high-risk localized prostate cancer.

**Methods and Materials:** From October 2009 to August 2012, 41 patients newly diagnosed, high-risk or very high-risk (NCCN definition) localized prostate cancer were treated with whole-pelvis irradiation and SBRT boost. The whole pelvis dose was 45 Gy (25 fractions of 1.8 Gy). The SBRT boost dose was 21 Gy (three fractions of 7 Gy). Ninety percent of these patients received hormone therapy. The toxicities of gastrointestinal (GI) and genitourinary (GU) tracts were scored by Common Toxicity Criteria Adverse Effect (CTCAE v3.0). Biochemical failure was defined by Phoenix definition.

**Results**: Median follow-up was 42 months. Mean PSA before treatment was 44.18 ng/ml. Mean PSA level at 3, 6, 12, 18, and 24 months was 0.94, 0.44, 0.13, 0.12, and 0.05 ng/ml, respectively. The estimated 4-year biochemical failure-free survival was 91.9%. Three biochemical failures were observed. GI and GU tract toxicities were minimal. No grade 3 acute GU or GI toxicity was noted. During radiation therapy, 27% of the patient had grade 2 acute GU toxicity and 12% had grade 2 acute GI toxicity. At 3 months, most toxicity scores had returned to baseline. At the last follow-up, there was no grade 3 late GU or GI toxicity.

**Conclusions**: Whole-pelvis irradiation combined with SBRT boost for high-risk localized prostate cancer is feasible with minimal toxicity and encouraging biochemical failure-free survival. Continued accrual and follow-up would be necessary to confirm the biochemical control rate and the toxicity profiles.

## Introduction

Prostate cancer is not only the most common male malignancy in the US (1) and the rest of the Western world but also a rising health problem in Asia (2, 3). It is worth noting that before or in the early PSA era (late 1980s and early 1990s) patients presented with higher volume disease than in the current PSA screening era (4, 5). However, the current estimates indicate that high-risk disease accounts for 15% of all prostate cancer diagnoses in the US (6) and even higher in the low-PSA screening regions, like Asia (7). In a Japanese institutional database, approximately 50% of prostate cancer patients had high-risk disease at diagnosis (8).

Generally, patients with high-risk prostate cancer have a significant chance of developing systemic or local recurrence and are at higher risk for symptoms or death from the disease (9). Patients of high-risk prostate cancer have a more aggressive form of the disease with the higher possibility of direct extension and/or locoregional micrometastases and require a more intensive approach of treatment to maintain a normal life expectancy.

According to the modern understanding of radiobiology, prostate cancer is more sensitive to large doses per fraction. Accumulating evidence has demonstrated that the α/β ratio of prostate cancer is lower than that of other common tumors and late-responding tissue (ranges from 1.2 to 3.1 Gy) (10–13); that is, using a hypofractionated radiotherapy schema may improve the biochemical control of prostate cancer without increasing toxicities associated with late-responding tissues (14).

The hypofractionated boost is used to supplement a course of conventionally fractionated external-beam radiotherapy (EBRT) designed to escalate the biological dose to a larger volume encompassing the microscopic disease adjacent to the prostate and seminal vesicles. High-dose-rate (HDR) brachytherapy using as a hypofractionated boost to EBRT has shown promising results (15–19).

The past few years have seen significant advances in radiotherapy techniques and stereotactic body radiotherapy (SBRT) is now at the forefront of innovation. The major features of SBRT are the accurate delivery of high doses to the target area and the rapid tapering of the dose away from the target area, using either a single or limited number of fractions. The SBRT boost in conjunction with EBRT in the treatment of prostate cancer is based on the clinical success of HDR brachytherapy boost, the modern understanding of radiobiology for prostate cancer, and advances in radiotherapy technique. Here, we report our early results of whole-pelvis radiotherapy (WPRT) and SBRT boost for high-risk localized prostate cancer.

## Material and Methods

### Patient selection

From October 2009 to August 2012, patients newly diagnosed with high-risk localized prostate cancer and treated with WPRT and SBRT boost were enrolled in this retrospective analysis. All patients had histologically confirmed primary adenocarcinoma of the prostate. Stage was determined by physical exam, bone scan, and MRI or CT scans. None of these patients had received any other local or systemic primary treatment of prostate cancer when enrolled in this treatment protocol, except neoadjuvant hormone therapy (NHT). Prior transurethral resection of the prostate (TURP) for urinary symptom relief was allowed. All the patients were classified as high-risk or very high-risk group, defined by National Comprehensive Cancer Network (NCCN) guidelines with the presence of any one of the following high-risk factors: pre-biopsy PSA ≥ 20 ng/ml, Gleason score 8–10, and clinical stage T3. The institutional review board approved this retrospective analysis.

### Radiotherapy preparation

Patients underwent transrectal implantation of four fiducials before WPRT, with two seeds placed at the prostate apex and two at the base. CT simulation was performed 1 week after fiducial implantation to account for possible fiducial migration. A custom vacuum cushion, knee support, and other devices were applied to all patients for immobilization. WPRT and SBRT boost treatment planning were based on the thin-slice CT images (1–2 mm in thickness). MRI fusion was utilized as a supplement for anatomical contour delineation.

### Whole-pelvis radiotherapy treatment planning and delivery

#### Target volume delineation

The prostate gland, the entire seminal vesicles (with or without invasion), and the area of radiographic extracapsular extension were defined as the clinical target volume (CTV) 1. CTV 2 included the four anatomical pelvic node groups (the external and internal iliac nodes, the presacral nodes, and the obturator nodes), following the RTOG consensus (20). The planning target volume (PTV) 1 was extended 7 mm beyond the CTV1 in all directions, except in the posterior direction, wherein it was extended 5 mm. The pelvic node PTV (PTV2) was extended 7 mm in all directions.

#### Organs at risk

The rectum was contoured as a solid organ from the bottom of the ischium to the sigmoid flexure. The entire bladder was contoured. The small bowel was contoured as the peritoneal cavity and the upper boundary was 1 cm higher than PTV2. For WPRT, the rectum constraint was less than 17% of the rectal volume to receive more than 42 Gy (V42 < 17%). Urinary bladder constraint was less than 40% of the urinary bladder volume to receive more than 40 Gy (V40 < 40%). Small bowel constraints were less than 0–1 cm^3^ of the small bowel volume to receive more than 52–54 Gy (V54–52 < 0–1 cm^3^) and mean small bowel dose less than 23.5 Gy (mean dose < 23.5 Gy).

#### Basic treatment plan criteria for WPRT

The prescription dose of WPRT was 45 Gy and was administered in 25 fractions. A minimum of 95% of the prescription dose was assured to cover 100% of the PTV.

**Figure 1:**
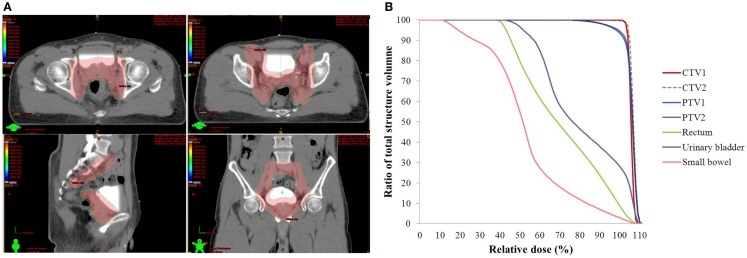
**Isodose curves (A) and dose–volume histogram (B) of whole pelvis radiotherapy for the applied RapidArc plan in the particular patient**. **(A)** The region of the prescription dose (45 Gy), pink color wash; CTV1, red solid line.

All WPRT treatment plans were generated on Varian Eclipse treatment planning system (version 8.6.10, Varian Medical Systems, Palo Alto, CA, USA) (Figures 1A,B). The optimization and dose calculation were similar to the previous description (21). WPRT was delivered by RapidArc technique with daily image-guidance (two arcs, Varian Clinac iX).

### SBRT boost treatment planning and delivery

#### Target volume delineation

The prostate gland, the entire seminal vesicles (with or without invasion), and area of radiographic extracapsular extension were defined as CTV1, which was the same as in the WPRT treatment plans. For SBRT boost, the planning target volume (PTV) was extended 5 mm beyond the CTV in all directions, except in the posterior direction, wherein it was extended 3 mm.

#### Organ at risk

The rectum, urinary bladder, and penile bulb were contoured. For SBRT boost, the rectum constraints were less than 1 cm^3^ of the rectum volume to receive more than 20 Gy (V20 < 1 cm^3^) and less than 17% of the rectal volume to receive more than 14.5 Gy (V14.5 < 17%). Urinary bladder constraints were less than 5 cm^3^ of the urinary bladder volume to receive more than 21 Gy (V21 < 5 cm^3^) and less than 25% of the urinary bladder volume to receive more than 14.5 Gy (V14.5 < 25%). Penile bulb constraint was less than 50% of the penile bulb volume to receive more than 16.5 Gy (V16.5 < 50%).

#### Basic treatment plan criteria for SBRT boost

**Figure 2:**
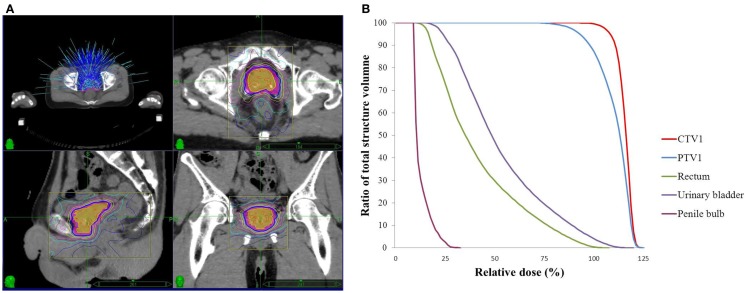
**Isodose curves (A) and dose–volume histogram (B) of SBRT boost for the applied CyberKnife in the particular patient**. **(A)** The prescription dose (21Gy), blue solid line; CTV1, orange color wash; PTV1, pink color wash.

The prescription dose of SBRT boost was 21 Gy in three fractions. A minimum of 95% of the prescription dose was made sure to cover 95% of the PTV after prescription to the 80% (or higher) isodose line. All SBRT boost treatment plans were generated on MultiPlan (version 2.2.0, Accuracy Incorporated, Sunnyvale, CA, USA) (Figures 2A,B). The optimization and dose calculation were similar to the previous description (21). SBRT boost treatments were delivered with CyberKnife (Accuracy). The SBRT boost began right after completion of WPRT and was administered in every-the-other days. Each treatment day, prior to SBRT, patients were asked to empty their bowels. Laxatives or glycerol ball enema were allowed.

### Hormone therapy

Hormone therapy was administrated at the discretion of the treating physician. In this study, LH-RH agonists, anti-androgen agents, or a combination were allowed for long-term use.

### Follow-up and toxicity assessment

All patients were scheduled for follow-up 4 weeks after final treatment, and then every 3 months thereafter. Acute and late toxicities of gastrointestinal (GI) and genitourinary (GU) tracts nature were assessed by Common Toxicity Criteria Adverse Effect (CTCAE version 3.0) at every follow-up visit. Acute toxicity was defined as occurring within 6 months of completing treatment, and late toxicity as those events occurring later than 6 months. PSA tests and self-administered IPSS quality of life (QoL) score questionnaires (22) were performed every 3 months. Biochemical failure was defined using the Phoenix definition. All post-treatment time intervals were calculated from the time of radiation therapy completion. Biochemical recurrence-free survival was estimated by Kaplan–Meier survival curve (SPSS, Version 19, IBM, USA).

## Results

### Patient characteristics and follow-up

A total of 41 consecutive patients were analyzed. The median age was 72.5 years (range, 61–83 years). Patient characteristics are summarized in Table 1. The median follow-up was 42 months (range, 1.5–58 months). One patient died from liver failure induced by hypersensitivity to anti-androgen agents. The other two patients died from distant metastasis after biochemical failures.

**Table 1:** **Patient characteristics**.

Characteristics	*n* (%)
Age (mean)	72.5 (range, 61–83)
**Primary t stage**
T1a–c	1(2.4%)
T2a–c	23(56.1%)
T3a	8(19.5%)
T3b	9(21%)
**Clinical nodal status**
N0	41(100%)
**Gleason score**
≤6	10(24.4%)
=7	14(34.1%)
≥8	17(41.5%)
**PSA level (ng/ml)**
Median	44.15 (range, 4.51–250.32)
≤10	7(17.1%)
10–20	9(21.9%)
>20	25(61.0%)
**NCCN risk group**
High-risk	32(78.1%)
Very high-risk	9(21.9%)
**Hormone therapy**
Neoadjuvant	22(53.7%)
Concurrent	16(39.0%)
No	3(7.3%)
**IPSS^a^ (Pre-treatment)**
0–7	13(33.3%)
8–19	16(41.0%)
20–35	10(25.7%)
**IIEF^a^ (Pre-treatment)**
0	21(53.8%)
≤21	38(97.4%)
≥22	1(2.6%)

*IPSS, international prostate symptom score; IEFF, international index of erectile function questionnaire*.

*^a^Only 39 patients completed the questionnaires*.

Twenty-eight patients (68.3%) received anti-androgen monohormone therapy, ten patients (24.4%) received LH-RH agonist-based hormone therapy, and three patients (7.3%) refused any hormone therapy.

### Toxicity profile and quality of life

#### Acute toxicity

**Figure 3:**
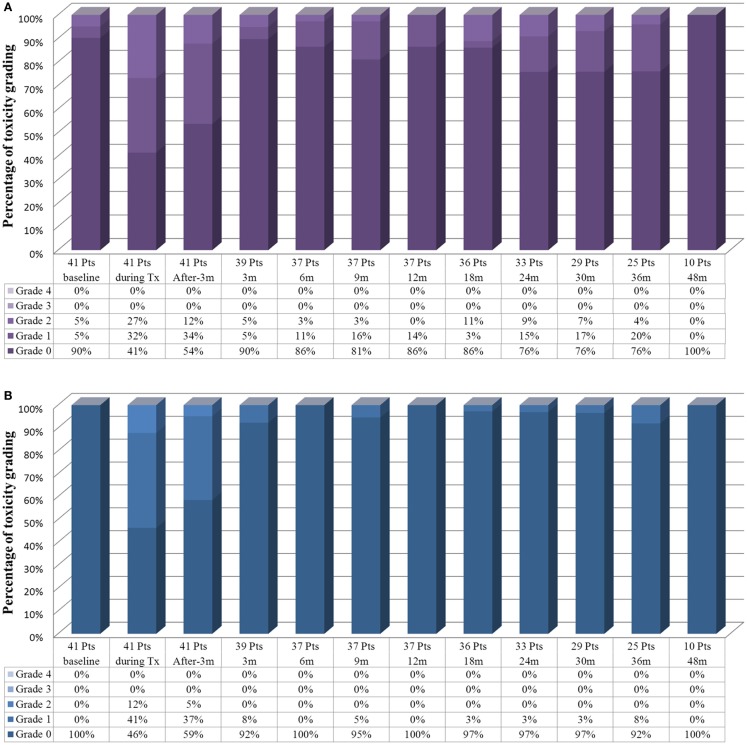
**Toxicity profile with time flame of the patients who underwent whole pelvis radiotherapy with stereotactic body radiotherapy boost**. **(A)** The genitourinary tract toxicity profiles and **(B)** the gastrointestinal toxicity profile.

Acute and late GI and GU toxicity profiles were minimal (Figure 3). The most common GU toxicities were frequency, urgency, and urinary obstructive symptoms. No grade 3 acute GU or GI tract toxicity was noted. During the radiation therapy, 27% of the patients had grade 2 acute GU toxicity and 12% had grade 2 acute GI toxicity. The course of the symptoms peaked at 1–2 weeks following the completion of the treatment. Most of toxicity scores had returned to baseline within 3 months.

#### Late toxicity

Fluctuating late GU grade 2 toxicities were observed in 3–11% of patients during the follow-up. There were no late grade 3 GU toxicity events. No late GI grade 2 or higher toxicities were observed at the last follow-up of the patients. Change in potency after radiotherapy was difficult to assess in the current study for two reasons. One was the impact of long-term hormone therapy. Most of the patients (97.4%) had no sexual activity or some erectile dysfunction before the treatment (Table 1).

#### Quality of life

In Table 2, we summarize the patient self-reported urinary QOL score at baseline, at 3 months, 1 year, and 2 years after completion of SBRT. Only one patient reported “terrible” (QOL score 6) urinary symptoms at 6 months. This patient was also “mostly dissatisfied/unhappy” (QOL score 4–5) at baseline. We noted that, although the urinary QOL scores deteriorated between 6th and 12th months, they recovered and in fact improved over baseline at 1 and 2 years, with more than 80% of the patients reporting QOL scores below 3.

**Table 2:** **Quality of life score**.

IPSS QoL score	Baseline (%)	1 month (%)	3 months (%)	6 months (%)	9 months (%)	12 months (%)	18 months (%)	24 months (%)
0–1	5.1	12.5	18.5	20.0	21.7	26.3	25.0	27.3
2–3	71.8	62.5	66.7	72.0	65.2	57.9	56.3	63.6
4–5	23.1	25.0	14.8	4.0	13.0	15.8	18.8	9.1
6	0.0	0.0	0.0	4.0	0.0	0.0	0.0	0.0

*IPSS QoL score, international prostate symptom score, quality of life score*.

### Biochemical failure-free survival and PSA response

**Figure 4:**
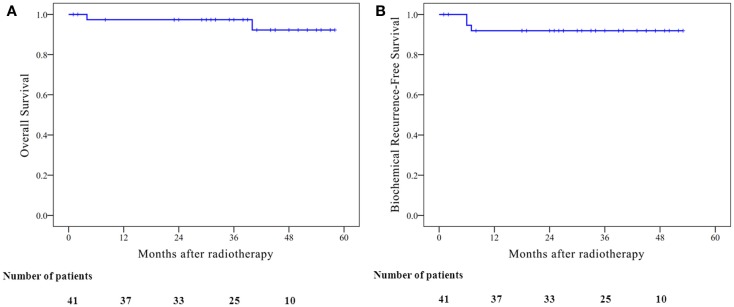
**Kaplan–Meier curves illustrate the survival of the patients who underwent whole pelvis radiotherapy with stereotactic body radiotherapy boost, including (A) overall survival; 4-year overall survival, 92.2% (B) biochemical recurrence-free survival; 4-year biochemical recurrence-free survival, 91.9%**.

Mean PSA level before the treatment was 44.18 ng/ml. Mean PSA level (excluding biochemical failures) at 3, 6, 12, 18, and 24 months was 0.94, 0.44, 0.13, 0.12, and 0.05 ng/ml, respectively. No obvious PSA bounce of >0.2 ng/ml was observed since long-term hormone therapy was applied. Only some mild elevation of PSA (>0.2 ng/ml) was observed after the cessation of long-term hormone therapy. The estimated 4-year overall survival and biochemical failure-free survival were 92.2 and 91.9%, respectively (Figure 4). Three biochemical failures were observed. All three biochemical failures occurred between 3 and 6 months post-treatment. Only one patient received prostate biopsy that showed negative founding, who then salvage successfully by weekly Taxane-based chemotherapy. Two other patients with bone metastasis evidence had not undergone prostate biopsy. The PSA level of these two patients continued to flare up to more 1000 ng/ml even under different systemic treatments; one patient died and one patient was lost.

## Discussion

### Encouraging results

The development of SBRT boost has been based on the clinical success of HDR brachytherapy boost. Several studies of HDR boost in conjunction of EBRT have reported 5-year biochemical control rates of 61–83% for high-risk prostate cancer (15–19, 23). HDR brachytherapy has led to a 3–10% late grade 3 GU toxicities and 3–7% of the patients who underwent HDR brachytherapy boost have experienced urethral stricture (15, 16, 18, 19, 23). Acute and late rectal toxicity rates have been minimal following HDR brachytherapy boost. Grade 1 and grade 2 late GI toxicities have been reported to be in the range of 2–17% (16, 18, 19, 23). Grade 3 or 4 late GI toxicities have been mentioned in the range of 0.5–1% (15, 19).

In the current study, the outcome of high-risk patients treated with WPRT, SBRT boost, and long-term hormone therapy was comparable to the above HDR boost series with the 4-year biochemical failure-free survival at 91.9%. There were no grade 3 or higher GU and no grade 2 or higher late GI toxicity events. And there were no urethral strictures at last follow-up.

Several studies have been published with use of SBRT as boost to WPRT in high-risk patients with the similar results (24–26). Therefore, SBRT boost is an attractive established option for dose escalation with some advantages to brachytherapy boost: (1) non-invasive procedure, (2) no requirement of transperineal catheter or urinary catheter, (3) no necessity for hospital admission, (4) ability to deliver high doses safely to extraprostatic or seminal vesicle invasion.

### Prostate-only SBRT for high-risk patients

Even though hypofractionation to the prostate only is now accepted as a therapeutic option for high-risk patients, (27) a single institutional data (28) showed no significant difference in the biochemical control rate (6-year biochemical disease-free survival, 69%) for high-risk patients either treated with prostate-only SBRT or WPRT with SBRT boost. Some 55.7% of the patients received androgen deprivation therapy (ADT). A pooled analysis of multi-institutional prospective trials (29) showed the 5-year biochemical relapse-free survival rate at 95, 83, and 78% for Gleason score ≤6, 7, and ≥8, respectively, and 95, 84, and 81% for low-, intermediate-, and high-risk patients, respectively. Only 38% of the high-risk patients were administered ADT.

Our results demonstrate a high biochemical control rate for high-risk patients, which is even higher than those with prostate-only SBRT or WPRT with SBRT boost studies. The possible reasons are (1) SBRT boost, (2)WPRT, and (3) long-term hormone therapy. This three-modality approach delivers such a potent therapy to the prostate, the extraprostatic extension, and seminal vesicles, thereby increasing the probability of eradicating all the local and regional disease and hormone therapy may play a role in eliminating occult systemic disease and enhancing synergistic effects to radiation.

### Rationale for pelvic node RT

Whether or not the elective irradiation of pelvic nodes provides any benefit as compared to treating the prostate only has been a longstanding therapeutic dilemma (30, 31). RTOG 94-13 initially showed that NHT with WPRT improved progression-free survival (PFS) and PSA relapse-free survival than prostate-only fields (30). The updated results of RTOG 94-13, however, showed diminished significance in the improvement in PFS with only a trend in favor of WP with NHT. These collective findings were interpreted to show an unexpected biologic interaction between the timing of hormone therapy and WPRT and concluded the biologic benefit of WPRT with NHT for high-risk prostate cancer (32). However, that conclusion might not be suitable to apply to the current status of prostate cancer risk distribution in PSA screening era (33).

On the other hand, two systematic reviews (34, 35) from surgical viewpoints have suggested that extended pelvic lymph-node dissection could improve survival by eliminating regional micrometastatic disease.

In the current study, most patients had high tumor burden (high PSA level, more advanced T stage, higher Gleason score), which might lead to higher rate of microscopic local/regional extension. Hence, WPRT was important in enhancing biochemical control rate in the current study.

### Comparing toxicity with a WPRT and SBRT boost study

In a WPRT and SBRT boost study (28), Katz et al. observed slightly higher GU toxicities in the WPRT with SBRT boost group than in the SBRT monotherapy group for high-risk patient (grade 2, 7.8 vs. 2.3%; grade 3, 3.9 vs. 2.3%). The WPRT with SBRT boost group also had a higher rate of grade 2 GI toxicity (13.3 vs. 0%).

We also had a similar observation comparing SBRT monotherapy for low- and intermediate-risk patients with WPRT and SBRT boost for high-risk patients in our institutional data (36). In the current study, we did not observe any acute or late grade 3 toxicity of either GI or GU tract. There were less than 11% late grade 2 GU toxicity and 0% of late grade 2 GI toxicity. This could be the result of the application of modern radiotherapy advances. We used volumetric modulated arc therapy technique for WPRT rather than 3D-conformal radiotherapy or 4-field box technique, followed by real time-tracking SBRT system. Hence, we were able to reduce the dose received by urinary bladder and rectum to prevent further toxicity (Figures 1 and 2). The advance of radiotherapy technology made WPBT effective and safe.

### Role of hormone therapy

The role of ADT with definitive radiotherapy for high-risk localized prostate cancer is well established from randomized clinical trials and meta-analyses (37–40). A meta-analysis (37) based on randomized clinical trials of RT alone vs. RT with ADT showed improvement in all survival outcome measurements. In another meta-analysis comparing short vs. longer duration of ADT, it concluded that a longer course of ADT was superior to the shorter course (38).

Taking into account the current stage migration from PSA screening, RT dose escalation, and the morbidities of long-term ADT (e.g., cardiovascular risk, endocrine detrition, QoL, depression, osteoporosis), there may be a little added therapeutic benefit from it (28, 29, 41).

However, since the patients in the current study are from a low-PSA-screening region and most of them had a higher tumor burden and the most attending physicians prescribed long-term hormone therapy for these patients. Most common regimen was anti-androgen agents, which is not a standard monohormone therapy agent considering the higher hepatotoxicity prevalence in Taiwan (42). The current NCCN Asia consensus stated that ADT was an effective treatment for prostate cancer patients with less significant comorbidities in Asia (43). Therefore, we shifted our hormone therapy regimen to short-term anti-androgen agents (2–6 months) in combination with long-term LH-RH agonists for 2 years.

All in all, our data contain the largest cohort of SBRT boost for high-risk disease in Asia, which showed excellent disease control rate and relatively low rate of acute and late toxicity profiles. But it should be cautioned that all these modalities might cause some side effects. How to modify and optimize these three modalities will depend on the results of the ongoing clinical trials.

In conclusion, whole pelvis irradiation combined with SBRT boost is an attractive treatment option for patients with high-risk disease, with the potential to increase biological dose and thus improve biochemical control, without increasing toxicity and, from a practical viewpoint, reducing overall treatment time. Continued accrual and follow-up would be necessary to confirm the biochemical control rate and the toxicity profiles.

## Author Contributions

Study concepts and study design: Yu-Wei Lin, Li-Ching Lin, and Kuei-Li Lin. Data acquisition: Yu-Wei Lin, Li-Ching Lin, and Kuei-Li Lin. Quality control of data and algorithms: Yu-Wei Lin, Li-Ching Lin, and Kuei-Li Lin. Data analysis and interpretation: Yu-Wei Lin. Statistical analysis: Yu-Wei Lin and Li-Ching Lin. Manuscript preparation: Yu-Wei Lin and Kuei-Li Lin. Manuscript editing: Yu-Wei Lin and Li-Ching Lin. Manuscript review: Yu-Wei Lin, Li-Ching Lin, and Kuei-Li Lin.

## Conflict of Interest Statement

The authors declare that the research was conducted in the absence of any commercial or financial relationships that could be construed as a potential conflict of interest.
